# Melanoma Biomolecules: Independently Identified but Functionally Intertwined

**DOI:** 10.3389/fonc.2013.00252

**Published:** 2013-09-24

**Authors:** Danielle E. Dye, Sandra Medic, Mel Ziman, Deirdre R. Coombe

**Affiliations:** ^1^School of Biomedical Science & Curtin Health Innovation Research Institute, Faculty of Health, Curtin University, Perth, WA, Australia; ^2^School of Medical Sciences, Edith Cowan University, Perth, WA, Australia; ^3^School of Pathology and Laboratory Medicine, University of Western Australia, Perth, WA, Australia

**Keywords:** melanoma, CD146, CSPG4, galectin-3, MMP2, Pax3, biomarker

## Abstract

The majority of patients diagnosed with melanoma present with thin lesions and generally these patients have a good prognosis. However, 5% of patients with early melanoma (<1 mm thick) will have recurrence and die within 10 years, despite no evidence of local or metastatic spread at the time of diagnosis. Thus, there is a need for additional prognostic markers to help identify those patients that may be at risk of recurrent disease. Many studies and several meta-analyses have compared gene and protein expression in melanocytes, naevi, primary, and metastatic melanoma in an attempt to find informative prognostic markers for these patients. However, although a large number of putative biomarkers have been described, few of these molecules are informative when used in isolation. The best approach is likely to involve a combination of molecules. We believe one approach could be to analyze the expression of a group of interacting proteins that regulate different aspects of the metastatic pathway. This is because a primary lesion expressing proteins involved in multiple stages of metastasis may be more likely to lead to secondary disease than one that does not. This review focuses on five putative biomarkers – melanoma cell adhesion molecule (MCAM), galectin-3 (gal-3), matrix metalloproteinase 2 (MMP-2), chondroitin sulfate proteoglycan 4 (CSPG4), and paired box 3 (PAX3). The goal is to provide context around what is known about the contribution of these biomarkers to melanoma biology and metastasis. Although each of these molecules have been independently identified as likely biomarkers, it is clear from our analyses that each are closely linked with each other, with intertwined roles in melanoma biology.

## Introduction

The incidence of cutaneous melanoma has risen faster than any other malignancy in Causasian populations in the last 30 years, making it a global health problem (1). Although some of this increase may be due to improved surveillance, early detection and changes in diagnostic criteria, most is considered to be linked to increased sun exposure. Fortunately, the majority of patients present with thin, localized melanoma which in most cases is curable by surgical resection (2, 3). However, because melanoma metastasizes very early in the disease process, approximately 3% of patients who present with lesions <0.75 mm thick, 15% with lesions between 0.75 and 1.00 mm, and 30% with lesions >2.00 mm develop metastatic disease and die within 10 years (4, 5). The prognosis is significantly worse for those patients who present with regional and distant metastases at diagnosis, with 10 year survival rates of 64 and 16% respectively (6).

These poor survival rates are a reflection of the two main challenges in the management of metastatic melanoma – (1) the inadequacy of current prognostic markers and (2) the lack of effective treatment options. Currently, prognosis is based on a small set of clinical and histological features, e.g., tumor thickness, level of invasion, and ulceration (7), which have limited predictive power for individual patients and no direct implications for personalizing treatment (8). Therefore, there is an urgent need for a prognostic tool that can triage patients into high and low risk of metastatic melanoma, particularly for patients with thin melanoma, who show significant heterogeneity in survival (9). This would enable high-risk patients to receive necessary follow-up and adjuvant treatment while minimizing the interventions received by low risk patients. Moreover, melanoma is refractory to standard treatments such as chemo- and radiotherapy (10, 11), and new therapies are either effective for a relatively short time, e.g., BRAF inhibitors (12), or have serious side effects, e.g., ipilimumab, an immune-modulating antibody that targets CTLA-4 on activated T lymphocytes and suppressor T regulatory cells (13, 14).

Clearly, there is a significant need for both new biomarkers and new therapeutic options in melanoma. Intuitively, a biomarker with high predictive value may also be a potential therapeutic target. However, the discovery of new biomarkers and development of new treatments is challenging, as one molecule on its own is unlikely to have sufficient predictive value to be an effective biomarker. Similarly, therapies targeting a single molecule will also lack efficacy. The complexity of the metastatic process suggests an accurate prognostic tool-kit will include additional biomarkers to the current histological features used, while an effective treatment will require simultaneous targeting of multiple steps in the metastatic pathway (15).

Recent systematic reviews by Gould Rothberg et al. (16), Schramm and Mann (8), and Tremante et al. (17) used REMARK criteria (REporting recommendations for tumor MARKer prognostic studies) (18) to select high quality studies investigating melanoma biomarkers. From these reviews and others we have identified five melanoma biomarkers consistently associated with melanoma progression – melanoma cell adhesion molecule (MCAM), galectin-3 (Gal-3), matrix metalloproteinase-2 (MMP-2), chondroitin sulfate proteoglycan 4 (CSPG4), and paired box 3 (PAX3). They comprise a transcription factor (PAX3), cell surface glycoproteins (MCAM and CSPG4), a secreted protein (Gal-3), and a matrix-degrading enzyme (MMP-2). These molecules were chosen because of their apparent involvement in different aspects of the disease process. Yet intriguingly, these five melanoma biomarkers are all linked by a network of overlapping functions in melanoma progression.

## Paired Box 3

PAX3/Pax3 (PAX3 and Pax3 represent the human and mouse factors respectively) is a member of the Pax family of transcription factors that are highly conserved throughout phylogeny. All play a crucial role in embryogenesis but all are also implicated in tumorigenesis – [for reviews see (19–21)]. Pax3 protein contains two DNA binding domains, a paired domain and a homeodomain which can be used alone or in combination to bind downstream target genes (22–25). In addition Pax3 contains a C-terminal transcription activation domain and an octapeptide (24, 26, 27). The ability of Pax3 to employ one or both DNA binding domains accounts for its ability to regulate numerous downstream targets. A single Pax3 gene encodes multiple transcripts produced by alternate splicing (28–31). The resultant protein isoforms provide functional diversity for Pax3, as they differ in structure and in the activity of their paired, homeodomain and alternate transactivation domains (31–33). Pax3 functions by activating or repressing expression of its downstream target genes, thereby affecting the target gene-mediated regulatory pathways. Moreover, certain protein modifications, e.g., acetylation, can switch Pax3 from an activator to a repressor on the same target gene promoter (34). In addition, different PAX3 isoforms seem to have a different (and even opposing) effect on the same cellular process (35).

PAX3 expression and function has been extensively studied in embryogenesis and its role here is well described [reviewed in Ref. (36)]. Its expression during early embryogenesis is critical for development of cells of neural crest origin, the cells that give rise to skin melanocytes. PAX3 is considered a key player in melanocyte development, from lineage specification and maintenance of melanoblast stemness, to regulation of cell proliferation and migration to their final location where they terminally differentiate into melanocytes (28, 37, 38). Pax3 is crucial for melanoblast specification and differentiation, being at the pinnacle of the hierarchy of melanocyte-specific gene regulators. In addition, Pax3, along with other factors, activates the key melanocytic regulator MITF (microphthalmia transcription factor) which initiates the activation of the cascade of melanogenic genes (39, 40). It is interesting to note that even though activation of Mitf by Pax3 during embryogenesis is well described, this regulatory axis does not seem to be operational in melanoma cells (41), where MITF and PAX3 regulate diverging pathways.

The involvement of PAX proteins in cancer is well known (20). Many studies show PAX3 expression in melanoma, but also in tumors arising from other neural crest-derived tissues, such as medulloblastoma, benign peripheral glial tumor neurofibroma (precursor of malignant nerve sheath tumor), Erwin’s sarcoma, supratentorial primitive neuroectodermal tumor, and pediatric alveolar rhabdomyosarcoma (ARMS) (42–51). In melanoma, PAX3 expression is evident at all stages of disease progression, including the primary lesion, circulating melanoma cells, and metastatic lesions (29, 42–46, 52–55). PAX3 is also expressed in benign naevi and in normal melanocytes (53, 56), although its precise role here is not clear. This suggests PAX3 is best described as a lineage marker rather than a marker of disease progression.

However, the recently proposed theory that melanoma progression is driven by those melanoma cells showing a highly motile, less differentiated (stem-like) phenotype (57–60), and the crucial roles PAX3 plays in melanocyte development, implies that it is more than just a lineage marker. It might actively drive melanoma progression. It has been suggested that the ability of a melanoma cell to respond to micro-environmental changes by switching between a highly proliferative (low metastatic potential, leading to tumor growth), and highly invasive phenotype (motile and stem cell-like, resulting in tumor dissemination) contributes to the aggressive nature of melanoma (60, 61). PAX3 is a nodal point in melanocyte differentiation, as it simultaneously functions to initiate the melanogenic cascade while preventing terminal differentiation, thus keeping the cell in a lineage restricted stem cell-like state (19). The evidence that PAX3 protein modifications, such as phosphorylation and acetylation, can alter cell functions, from stem-like to differentiated (34, 62–64), strengthens this hypothesis.

PAX3 has been shown to prevent apoptosis in melanoma cells (56, 65) via a range of mechanisms. Several known anti-apoptotic factors, such as tumor suppressors p53, PTEN, and Bcl-Xl, are mediators of Pax3-induced cell survival, in both embryogenesis and tumorigenesis. Pax3 has a dual effect on p53; it represses transcription of p53-dependant genes, *BAX* and *HDM2-P2*, and promotes p53 protein degradation (66). Knock down of PAX3 induced increased cell detachment, growth reduction, and increased apoptosis in melanoma cell lines (65). Inactivation of the tumor suppressor gene PTEN is often found in PAX3-positive tumors (67). PAX3 binds directly bind to the *PTEN* promoter (68), down regulating its expression and decreasing apoptosis (69). PTEN regulates progression through the G1 cell cycle checkpoint, by negatively regulating PI3K/AKT signaling. Transcription of *BCL-XL*, a member of the *BCL-2* family of anti-apoptotic genes, is also directly regulated by PAX3 (68, 70). Treatment with *PAX3* or *BCL-XL* antisense oligonucleotides, individually or in combination, decreased cell viability to a similar extent, suggesting that *PAX3* and *BCL-XL* lie in the same anti-apoptotic pathway (70). Additionally, MITF regulates another member of the same gene family, *BCL-2* (71), providing an alternative indirect mechanism to regulate melanoma cell survival.

During embryogenesis Pax3 plays a crucial role in controlling the correct migration of cells, by directly regulating the transcription of TGFα and TGFβ (72, 73), growth factors that are involved in remodeling the extracellular matrix (ECM) and cell cytoskeleton as required for cell migration (73–75). A similar role is suspected in melanoma cells, where PAX3 has been found to directly target the *TGF*β promoter in metastatic melanoma cell lines (68). Involvement of PAX3 in melanoma migration is further supported by evidence showing that other genes associated with cell migration, including *MCAM*, *CSPG4*, and *CXCR4*, are targeted by PAX3, as shown by ChIP assay in A2058 melanoma cells (68). Up-regulation of MCAM expression following *Pax3*-transfection in melanoma cells confirmed that MCAM is a downstream target of Pax3 (76, 77), and the number of cells co-expressing MCAM and PAX3 is increased in highly metastatic melanoma (53). CXCR4 is also associated with metastatic spread of melanoma (78). CXCR4, and its ligand CXCL12, regulates chemotactic migration and “homing” of tumor cells to a secondary organ/site, and facilitates tumor cell extravasation (79, 80).

Medic et al. (68) suggested the traditional developmental roles of PAX3 in regulating differentiation, proliferation, cell survival, and migration, are retained in melanoma cells. They showed that PAX3 promoted a less differentiated, stem-like (via HES1, SOX9, NES, DCT), motile (via MCAM, CSPG4, and CXCR4) phenotype, characteristic of melanomas with high metastatic potential (81). PAX3-mediated regulation of melanoma cell survival and proliferation is through BCL2L1 and PTEN, and TPD52 (tumor protein D52) respectively (82). By controlling crucial cell processes (proliferation, cell survival, and migration), as well as promoting a less differentiated stem-like phenotype, PAX3 “ticks all the boxes” as an intrinsic factor driving melanoma development and progression.

From these studies it is evident that PAX3 is involved in melanoma progression on multiple levels, and it is likely that at different stages of disease progression, PAX3 plays different roles. Most recently, PAX3 has been identified as the mediator of anti-senescence and induced drug resistance in melanoma cells (83–85). Consistent with its crucial roles in normal melanocytes and melanoma cells, PAX3 appears to be expressed on similar percentages of circulating tumor cells (CTCs) in patients with different stages of metastatic disease (AJCC stages 0–V). However, this percentage decreased in patients following surgical removal of metastatic lesions, suggesting PAX3 expression could be used to monitor the tumor load in patients undergoing surgery and other treatments (55).

## Melanoma Cell Adhesion Molecule

Melanoma cell adhesion molecule (CD146, Muc18, S-Endo-1) is a cell surface glycoprotein belonging to the immunoglobulin (Ig) superfamily. It has five extracellular Ig-like domains, a short transmembrane region, and a cytoplasmic tail, which includes two putative endocytic motifs (86, 87). MCAM was initially identified as a marker of melanoma progression in 1989 (88), and recently was recognized as a more accurate prognostic marker than all other clinico-pathological characteristics (89). MCAM is expressed on approximately 70% of primary melanoma and 90% of lymph node metastases, and MCAM expression in a primary lesion is predictive of lymph node metastases and metastases at other sites (90). MCAM expression is also associated with significantly lower 5 year survival rates: approximately 95% of patients with MCAM negative primary lesions survive 5 years post-diagnosis, compared to 40% of patients with MCAM positive primary lesions. Stratification of patients by MCAM expression in the primary tumor may therefore enable more accurate identification of patients who are likely to have a positive lymph node, and those patients that have high-risk of recurrence despite a negative lymph node (90).

In addition to melanoma, MCAM expression has been linked to progression of breast, prostate, and ovarian cancer (91–95). Interestingly, MCAM also plays a role in trophoblast invasion during pregnancy (96, 97) and is used as a marker of mesenchymal stem cells (98, 99). In normal adult tissue, MCAM is primarily expressed by the vascular endothelium and smooth muscle (100, 101). Most studies on MCAM have focused either on its contribution to melanoma metastasis or its role in endothelial cell function and angiogenesis.

On melanoma cells, MCAM mediates cation independent cell–cell adhesion (102), moderates cell-matrix interactions (103) and is associated with increased cell migration and invasion, as seen in *in vitro* scratch wound and invasion assays (104, 105). A blocking antibody to MCAM decreased cell–cell adhesion and cell invasion *in vitro*, and decreased primary tumor growth and lung metastases *in vivo* (106). Other murine studies suggest MCAM influences the later stages of metastasis, such as the establishment of a secondary lesion (107). In endothelia, MCAM has been implicated in maintenance of endothelial cell–cell junctions (101, 108), endothelial cell proliferation, migration, and angiogenesis (109).

Data from human studies also suggest that MCAM expression may be linked to the development of metastatic melanoma lesions. MCAM expression on CTCs in melanoma patients has been associated with increased tumor burden and poorer outcome in Stage IV disease (55, 110). In addition, MCAM expression on CTCs was found to be a useful marker for monitoring response to therapy, as patients with poor outcomes had an increased incidence of MCAM positive CTCs compared to patients with more positive outcomes (55, 110). Reid et al. (55) also suggest that MCAM expression on CTCs may help identify patients that respond poorly to conventional treatments and may benefit from alternative regimes. Despite the overwhelming evidence that MCAM expression on a melanoma lesion is associated with a poor prognosis, details of the key molecular interactions in melanoma progression that involve MCAM remain unclear. We, and a small number of other groups, have been exploring how the structural features of MCAM contribute to its role in melanoma progression as a way of redressing this issue.

Melanoma cell adhesion molecule has eight potential N-glycosylation sites (88) and is heavily glycosylated during post-translational processing, with approximately 35% of its weight due to carbohydrate modifications (111). Sialic acid, the HNK-1 antigen (CD57), and β1–6 branched *N*-acetylglucosamine side-chains (β1–6 branches) (111) are among the carbohydrates moieties carried by MCAM, although the carbohydrate structures decorating MCAM vary according to the cell-type which is expressing this protein. MCAM exists as monomers and dimers on the surface of both endothelial and melanoma cells (112); with dimerization mediated through a disulfide bond occurring between cysteine residues in the most membrane proximal Ig domain (113). There are two isoforms of MCAM: MCAM-long contains a 63 amino acid intracellular domain including two putative endocytic domains and five potential protein kinase recognition sites (100), while MCAM-S contains a truncated cytoplasmic tail that lacks both of the endocytosis motifs and one of the protein kinase sites (87). Melanoma cells express primarily the long isoform whereas endothelial cells express both (87, 103). A soluble form of MCAM has also been detected in cell culture supernatants and serum from normal healthy subjects (114).

The intracellular tail of MCAM-long binds to hShroom1 (87) and ezrin-radixin-moesin (ERM) proteins (115), both of which bind to the actin cytoskeleton. Luo et al. (115) found that the ERM proteins link MCAM to the actin cytoskeleton and promoted the formation of microvilli. In addition, the MCAM-ERM protein complex recruited Rho guanine nucleotide dissociation inhibitory factors 1 (RhoGDI1) and sequestered it from RhoA. The release of RhoA from RhoGDI1 inhibition led to RhoA activation, downstream signaling and widespread microfilament reorganization (115). Activation of the PI4P5K-PIP_2_ pathway during this process formed a positive feedback loop, further promoting the phosphorylation and activation of the ERM proteins and the MCAM-ERM interaction (115). The regulation of cytoskeletal reorganization and migration by RhoA in melanoma cells in response to the chemokine CXCL12 (SDF-1), has previously been described (116, 117). Thus, Luo et al. (115) proposed the overexpression of MCAM in melanoma cells drives RhoA activation, cytoskeletal reorganization, and cell migration.

Witze et al. (118) describe a different model for the contribution of MCAM to cell polarity and migration of melanoma cells. They described Wnt5-mediated recruitment of MCAM, actin, and myosin IIB into intracellular bodies known as Wnt5a-mediated receptor-actin-myosin polarity (W-RAMP) structures. In the presence of CXCL12, these structures distributed asymmetrically and directed membrane retraction at the trailing edge of the cell. Membrane retraction then promoted nuclear movement and influenced the direction of cell migration (118). This process required membrane internalization, endosomal trafficking, and the intracellular translocation of MCAM, and in contrast to other Wnt-cytoskeletal interactions and the model proposed by Luo et al. (115) it is regulated by RhoB rather than RhoA.

Endothelia and melanoma express high levels of MCAM, and as melanoma cell interactions with vascular endothelia are a key part of the metastatic process, it is likely MCAM on both of these cells contributes to melanoma metastasis. Although a homophilic interaction between MCAM cannot be demonstrated (102, 119), it is possible that melanoma and endothelial cells both express MCAM and its ligand, and these interact bi-directionally. It is known that MCAM contributes to cell–cell adhesion in the vascular endothelium (108) and that engagement of the extracellular domain of MCAM initiates outside-in signaling resulting in calcium flux and the phosphorylation of a panel of intracellular proteins, including p125^FAK^ and paxillin, which leads to focal adhesion formation (120). Collectively, these data suggest the localization and function of MCAM at endothelial cell junctions involves dynamic interactions with, and reorganization of, the actin cytoskeleton (121). There is also evidence that MCAM expression in melanoma cells modulates the expression (103) and/or activity of integrin chains. The most compelling evidence involved the β1 chain. MCAM overexpression also appears to stimulate the expression of MMP-2. The association of MCAM with MMP-2 expression was first reported in the late 1990s (106, 122, 123). A recent study further revealed that MCAM is involved in signaling cascades that affect the expression of the transcriptional regulator, inhibitor of DNA binding-1 (Id-1) and activating transcription factor (ATF)-3 (124). This study showed that MCAM silencing increased the expression of ATF-3 and decreased the expression of Id-1. Interestingly, Id-1 expression was shown to positively regulate MMP-2 transcription. As AFT-3 binds to the Id-1 promoter and represses its transcription, the suggestion was that MCAM indirectly led to an increase in MMP-2 levels via decreasing AFT-3 and increasing Id-1 levels (124). These examples illustrate that MCAM expression may shift the balance between cell–cell and cell-matrix adhesion, in addition to increasing migration and invasion via the up-regulation of pro-invasive enzymes.

Jiang et al. (125) showed that MCAM interacts with vascular endothelial growth factor receptor 2 (VEGFR-2) on endothelia and acts as a co-receptor for the binding of vascular endothelial growth factor A (VEGF-A). The interaction of the extracellular domain of MCAM with VEGFR-2 occurred independently of VEGF-A, and was a crucial step in VEGFR-2 activation. When associated with VEGFR-2, the cytoplasmic tail of MCAM recruited ERM proteins and the actin cytoskeleton, to assemble a “signalosome,” which was required for signal transduction from VEGFR-2 to AKT and P38 MAPKs. The result was increased endothelial cell migration (125). MCAM can also function independently of VEGFR-2, and VEGF-A (109, 113). The interaction of MCAM with VEGFR-2 on melanoma cells remains to be confirmed, although it is known melanoma express VEGF and VEGFR-2, and overexpression of VEGF-A in a melanoma cell line with VEGFR-2 favored cell growth and survival through MAPK and PI3K signaling pathways (126).

Laminin 411 (laminin 8) and galectin-1 (Gal-1) have also been described as ligands for MCAM (127, 128). Flanagan et al. (128) reported that MCAM expressed by a subset of CD4+ T-cells (Th17 cells) binds laminin 411 from the vascular endothelia and this interaction was blocked by an anti-MCAM antibody and soluble recombinant MCAM (MCAM-Fc). Animal studies showed that an anti-MCAM antibody administered *in vivo* reduced Th17 lymphocyte infiltration into the central nervous system. The interaction of MCAM with laminin 411 is consistent with the interaction of gicerin (the avian homolog of MCAM) with neurite outgrowth factor, a member of the laminin family (129, 130), and basal cell adhesion molecule (an immunoglobulin superfamily member) with laminin 511 (131). The interaction of MCAM on melanoma with laminin 411 has not been investigated, but it is known that MCAM does not interact with laminin 111 (105), 511, or 332. The interaction of MCAM with Gal-1 is carbohydrate mediated. Gal-1 is produced by vascular cells and binds to carbohydrates on cell surfaces and ECM proteins (132). It has been implicated in angiogenesis (133) and melanoma progression and Jouve et al. (127) hypothesized that the interaction of MCAM with Gal-1 protects cells from Gal-1 induced apoptosis.

In conclusion, MCAM expression in a primary melanoma appears to increase the likelihood of metastatic spread and may assist to stratify patients into low and high-risk of recurrence at diagnosis (90). In addition, it is also useful as a marker on CTCs, as MCAM-expressing CTCs appear to correlate with tumor burden and disease progression (55). In melanoma, MCAM appears to facilitate cell migration by the rearrangement of the cellular cytoskeleton via activation of Rho proteins (115, 118), and potentially via activation of the AKT and P38 MAPK pathway in association with VEGRF (125). MCAM expression is also correlated with up-regulation of MMP-2 (124), and a modulation of integrin-mediated cell spreading and migration.

## Galectin-3

Galectin-3 belongs to a family of lectins that bind β-galactosides. It is found in the nucleus, cytoplasm, and on the cell surface of many cell types, and is also secreted into the extracellular space. It has a C-terminal carbohydrate recognition domain (CRD) and an N-terminal tail that mediates the oligomerization of Gal-3 molecules, which is vital for its extracellular functions (134). Gal-3 also contains an amino acid motif, NWGR, which is involved in its anti-apoptotic function. This motif is also found in Bcl-2 and has been called an “anti-death” motif. Like Bcl-2 family members Gal-3 exerts its anti-apoptotic activity at the peri-nuclear mitochondrial membranes (135). Extracellular Gal-3 binds with high affinity to *N*-acetyllactosamine containing glycans and binds to both cell membrane and ECM proteins that carry these glycosylation structures. Gal-3 binds a host of membrane proteins including integrins (e.g., β1, αv, αM), cell adhesion molecules (e.g., N-cadherin, NCAM, VCAM), lysosomal membrane associated glycoproteins (Lamps)-1 and -2, growth factor receptors (e.g., epidermal growth factor receptor, transforming growth factor β receptor), and molecules associated with the immune response including the T lymphocyte receptor (136, 137). Its ECM protein ligands include laminins-111, -332, -511, fibronectin, collagen IV, vitronectin, and elastin (137). The N-terminal domain of Gal-3 can be post-translationally modified via phosphorylation at Ser 6. Phosphorylation of this site influences the intracellular distribution of Gal-3 and therefore its ability to regulate transcription of downstream genes, anti-apoptotic functions, and carbohydrate binding properties. Specifically, phosphorylation is required for Gal-3’s anti-apoptotic function, and dephosphorylation for realization of its full ability to bind carbohydrate ligands (138).

Galectin-3 is expressed in the nucleus, cytoplasm, and plasma membrane of melanoma cells (139). The intra- and extracellular distribution of Gal-3 and its variety of extracellular binding partners, both on the cell surface and in the tumor microenvironment, suggests Gal-3 could affect metastatic progression via a range of mechanisms (139).

There is a growing literature indicating Gal-3 expression is associated with tumor progression in melanoma. Consistently the data indicate primary melanomas express significantly more Gal-3 than naevi (140–142). Gal-3 expression has also been positively correlated with tumor thickness, Clarke and Breslow tumor stage, lymphatic invasion, lymph node positivity, and distant metastases (143), although Brown et al. (144) recently reported that Gal-3 expression showed a bi-modal distribution, with increased levels in thin primary melanoma compared to naevi, and a progressive decrease in expression in thicker and metastatic melanoma. The decrease in Gal-3 expression in metastatic melanoma was particularly evident in the nucleus (144). This bi-modal distribution of Gal-3 was also reported by Vereecken et al. (142). Brown et al. (144) suggest that high Gal-3 in thin melanoma may contribute to resistance to apoptosis (145), but as a lesion progresses, intracellular Gal-3 may be released by the cell into the extracellular environment. Once in the extracellular environment, Gal-3 can interact with cell surface and ECM proteins. Melanoma progression may be associated with a decrease in intracellular stores of Gal-3, such that a decrease in Gal-3 expression may be associated with metastatic spread and a worse prognosis in melanoma (144). Curiously Gal-3 expression was reported to vary depending on the extent to which the melanoma lesion was exposed to the sun, chronically sun-exposed melanoma displayed nuclear Gal-3, whereas melanomas on intermittently sun-exposed sites had cytoplasmic staining for Gal-3. The authors of this study concluded that UV light may be involved in Gal-3 activation and that the translocation of Gal-3 to the nucleus is associated with a more aggressive lesion (140). The prognostic significance for melanoma of serum Gal-3 has also been investigated. This work suggested Gal-3 could be of prognostic value, as American Joint Committee on Cancer (AJCC) stage 3 and 4 melanoma patients had higher serum Gal-3 levels than patients with AJCC stage 1 and 2 melanoma, and serum measurements could have a role in follow-up and management of stage 3 and 4 melanoma patients (146).

Nuclear Gal-3 contributes to melanoma metastasis by regulating multiple genes such as VE-cadherin, MMP-1, MMP-2, interleukin 8 (IL-8), and autotaxin (135, 147–150). Wang et al. (150) reported that Gal-3 directly interacts with the transcription factor activating protein 1 (AP-1) to increase expression of MMP-1, which breaks down the collagens, types I, II, and III, thus enabling the migration of melanoma cells through interstitial connective tissue. In addition, Gal-3 expression in melanoma has also been associated with increased levels of VE-cadherin and IL-8, both of which are implicated in angiogenesis though the stimulation of vascular endothelial cell proliferation and migration. Gal-3 induced up-regulation of IL-8 has also been associated with increased MMP-2 expression (151). Recently, silencing Gal-3 expression in melanoma was shown to reduce expression of the transcription factor NFAT1 and so decrease the transcriptional activation and expression of autotaxin (lysophospholipase D) (149). Autotaxin was first identified from a human melanoma cell line due to its chemotactic and motility activity for melanoma cells (152). Autotaxin catalyzes the conversion of lysophosphatidylcholine (LPC) to lysophosphatidic acid (LPA), which acts as ligand for a range of G-protein coupled receptors to induce downstream signaling associated with migration, invasion, and angiogenesis in a range of cancers (153, 154). In melanoma, decreased autotaxin lowers melanoma growth and metastasis as well as affecting cell motility.

Gal-3 is also believed to play a role in the organization of cell membrane micro-domains. The cell membrane is a dynamic structure, with proteins clustered in non-random, functional domains held together by cohesive forces between proteins and lipids (155, 156). Most cell-surface proteins are glycosylated and oligomeric lectins such as Gal-3 bind to specific glycan structures on cell surface glycoproteins and help organize proteins into functional groups on the cell membrane (157, 158). These galectin-protein lattices are thermodynamically stable due to multiple low-affinity interactions, but are modulated by changes in protein glycosylation or galectin expression (159). Fluorescence recovery after photobleaching (FRAP) experiments revealed Gal-3 lattices on endothelial cells are stable and resistant to lateral movement once the Gal-3 oligomers have been formed (160). Further work has indicated Gal-3 lattices contribute to cell proliferation, migration, and apoptosis (155). By stabilizing glycoproteins in the cell membrane, Gal-3 lattices reduce receptor endocytosis (161) and influence the turnover of focal adhesions (162). Goetz et al. (162) found that Gal-3 lattices promoted integrin clustering, and with Caveolin-1 tyrosine phosphorylation, this stabilized focal adhesion kinase (FAK), paxillin, and α5 integrin in focal adhesion (FA) complexes. This decreased the exchange of FA components with the cytosol and facilitated FA maturation and turnover. The control of FA dynamics is critical for cell motility, as the assembly, maturation, translocation, and disassembly of FAs mediate cell attachment, contraction, protrusion of the leading edge, and retraction of the trailing edge during cell migration (163). Saravanan et al. concluded from their experiments with epithelia that on these cells Gal-3 cross-linked and clustered α3β1 integrins at the leading edge of migrating cells. Integrin clustering activated FAK and Rac1, which promoted lamellipodia formation and cell migration (164). We are currently performing experiments with melanoma cells to determine whether this model also holds for melanoma cell migration.

In addition to binding to cell and matrix components, Gal-3 is also cleaved by MMP-2 and MMP-9 to produce a biologically active fragment that that may be involved with cell invasion (147) and angiogenesis. These enzymes cleave extracellular Gal-3 to separate the C-terminal CRD from the N-terminal domain. Curiously, the 22-kDa cleaved fragment containing an intact CRD was found to bind its glycan ligands more strongly than the intact protein, under conditions when the concentration of the intact protein is such that oligomerization is prevented (147). Moreover, the data suggested that truncated Gal-3 effectively competes with full length Gal-3 to inhibit its homophilic cross-linking and other types of protein–protein interactions as treatment with the truncated form showed reduced tumor growth and metastasis in a breast cancer model (165).

Exogenous Gal-3 (secreted by melanoma cells) could also influence melanoma progression as a result of its role in angiogenesis. Gal-3 been shown to stimulate capillary tube formation of endothelial cells *in vitro* and angiogenesis *in vivo* (166). Interestingly the angiogenic activity of Gal-3 involves CSPG4 and the integrin α3β1. The binding of soluble CSPG4 to endothelial cell surfaces induced cell motility and the formation of a multicellular network on type I collagen gels. Antibody blocking studies indicated that both Gal-3 and α3β1 were involved in CSPG4 endothelial cell motility and that these molecules formed a complex on the endothelial cell surface (167). CSPG4 is expressed by microvascular pericytes whereas, Gal-3 and α3β1 are expressed by vascular endothelial cells, but as the regulation of the development of new vessels involves cross-talk between pericytes and endothelial cells it is likely that the signaling complex of α3β1, Gal-3, and CSPG4 is involved in pericyte endothelial cell cross-talk during early stage angiogenesis (167). Vascular endothelial expressed Gal-3 was also shown to important for the adhesion of melanoma cells to lung endothelia, which led to the suggestion that Gal-3 on lung endothelia could serve as the first anchor for circulating melanoma cells undergoing extravasation (168). Oligomerization of Gal-3 on endothelial cells to form lattices has been observed experimentally, with most Gal-3 concentrated in the cell–cell junctions. Fluorescent energy transfer (FRET) experiments with neutrophil adhesion suggested that oligomerized Gal-3 mediated neutrophil adhesion to endothelial layers primarily at the endothelial cell–cell junctions (160). It is very likely that melanoma cells similarly interact with endothelial cells via Gal-3 lattices. This conclusion is supported by Gal-3 knock-out studies that revealed Gal-3^−/−^ mice were resistant to lung melanoma metastases and melanoma cells bound less well to lung tissue from Gal-3^−/−^ mice (169).

The involvement of the immune system in checking melanoma progression has been an avenue for exploration for many years. It now seems that Gal-3 expression contributes to the effectiveness of leukocyte interactions with melanoma. A melanoma biopsy study reported a correlation between Gal-3 expression and the level of apoptotic tumor-associated lymphocytes (170).

The studies reviewed here indicate that Gal-3 is involved in many aspects of melanoma progression. Nuclear Gal-3 has been implicated in melanoma cell proliferation (probably in the earlier stages), while secreted Gal-3 in the tumor microenvironment has been linked to migration and invasion of melanoma cells and angiogenesis. Thus, the location of Gal-3 as well as the overall levels of Gal-3 expression could be useful as a biomarker or prognostic indicator at different stages of melanoma progression.

## Chondroitin Sulfate Proteoglycan 4

Chondroitin sulfate proteoglycan 4 (CSPG4) was first identified over three decades ago as a surface antigen on human melanoma cells (171). This molecule has been variously named high molecular weight melanoma associated antigen (HMW-MAA), melanoma chondroitin sulfate proteoglycan (MCSP), and nerve/glial antigen 2 (NG2), the latter originally identified on rat glia. CSPG4/NG2 positive cells make up about 5–10% of glia in the developing and adult central nervous system and these cells are believed to comprise a progenitor population, which matures into oligodendrocytes and subpopulations of astrocytes. Immature Schwann cells of the peripheral nervous system also express CSPG4/NG2 (172) as do pericytes in newly formed blood vessels (173), and cells of mesenchymal lineages, such as immature chondrocytes, osteoblasts, and myoblasts. In addition, cells in the basal layer of human epidermis and in the outer root sheath of hair follicles that co-express CSPG4 and high levels of β1-integrin are interfollicular epidermal stem cells and the numbers of these cells decrease with age (174, 175). CSPG4 has thus been called a stem cell marker.

CSPG4 is a single pass type I membrane glycoprotein. The intact core protein of 250 kDa has a large extracellular domain which consists of three structural domains: (1) a globular domain of two laminin G-Type regions, (2) a central region of 15 CSPG4/NG2 repeats containing 7 Ser-Gly motifs, one of which is the consensus motif SGXG for glycosaminoglycan attachment, and (3) a membrane proximal globular domain (D3) that contains 6 of the 15 potential sites for N-linked glycosylation. This domain also contains a number of possible proteolytic cleavage sites; cleavage here would give rise to soluble CSPG4 that can be detected in sera. The first globular domain has a compact configuration containing 8 of the 10 extracellular cysteines and 3 potential N-linked glycosylation sites. The 76 amino acid cytoplasmic domain contains threonines that can be phosphorylated by PKCα and ERK 1,2 (residues 2256 and 2314, respectively); a proline rich region that may contain a non-canonical Src Homology type 3 (SH3) domain binding motif, and a C-terminal 4 residue PDZ binding motif (176, 177) that binds to the PDZ domain of scaffold proteins like syntenin and MUPP1 (178, 179). Despite its name, CSPG4 can be expressed without a covalently attached chondroitin sulfate chain making it a “part-time” proteoglycan. As the presence of the chondroitin sulfate chain affects the cell surface distribution of CSPG4 and various functions of the glycoprotein, it has been suggested that regulation of chondroitin sulfate chain attachment may be a way tumor cells control CSPG4 activities (176).

Like MCAM, CSPG4 is widely expressed on melanoma cells, appearing on >85% of cutaneous melanoma lesions and melanoma cell lines (180, 181). This antigen can distinguish metastatic melanoma cells in sentinel lymph nodes by immunohistochemistry and qRT-PCR assays, and CSPG4 is more sensitive and more specific than MART-1, a commonly used melanoma marker (182). The level of CSPG4 expression is similar between lentigo maligna, nodular, and superficial spreading melanoma lesions but it is lower in primary acral lentiginous melanoma lesions. Recent data indicate that approximately 54% of primary acral lentiginous melanoma lesions express the antigen and staining levels are generally weak (183). CSPG4 is, however, a sensitive marker for desmoplastic melanoma; 95% of desmoplastic primary lesions stained for CSPG4, and 86% of nodal metastases were CSPG4 positive (184). When qRT-PCR was used for diagnosis, CSPG4 mRNA was detected in metastatic desmoplastic lesions that did not express MART-1 (184). The use of CSPG4 in diagnosis of desmoplastic melanoma could potentially be very useful, as these lesions display unusual spindle cell morphology and lack the common clinical and histological characteristics of cutaneous melanoma, which complicates diagnosis. CSPG4 immunoreactivity is also an important diagnostic indicator in the two forms of ocular melanoma (conjuctival and uveal). CSPG4 expression levels clearly separate conjuctival melanoma from conjuctival nevi and in one study lower CSPG4 expression appeared to be correlated with increased risk of recurrence (185). Most uveal melanoma also stain for CSPG4, with normal retinas and choroid displaying low immunoreactivity. CSPG4 may also be detected in the serum of some melanoma patients, but is not a reliable predictor of melanoma as only 29% of 117 melanoma patients had elevated serum CSPG4 (186). Immunomagnetic selection of CTCs from peripheral blood using antibodies to CSPG4 has been performed by a number of groups, using either one antibody or an antibody cocktail that recognizes different epitopes of CSPG4 (187–191). This method appears effective in enriching for circulating melanoma cells from peripheral blood samples. Collectively these studies provide convincing evidence that CSPG4 is a useful biomarker for melanoma.

Useful biomarkers generally have functions that aid either the initial development of the primary lesion or progression to metastases. The functions of CSPG4 could contribute to both of these processes. A number of reports have indicated that CSPG4 expression enhances the proliferation of melanoma cells *in vitro* and *in vivo*. This is true for murine melanoma cells (B16F1 and B16F10) transfected with NG2 and human melanoma cells (M14 and WM1552C) transfected with CSPG4 (192, 193). CSPG4 expressing WM1552C cells were also capable of anchorage-independent growth *in vitro* and had activated extracellular signal-regulated kinase (Erk)1,2, activities that required the cytoplasmic domain of CSPG4. Inhibition of CSPG4 expression by siRNA in melanoma cells expressing endogenous CSPG4 reduced Erk1,2 activation and anchorage dependent growth (193). Constitutive activation of the Erk1,2 pathway is associated with more advanced melanomas and the results of activation include entry into the cell cycle and increased expression of key melanoma transcription factors. CSPG4 can bind to and present growth factors, like FGF-2 and PDGF-AA, that impact on the Erk1,2 pathway. Although many advanced melanoma present with a mutation in BRAF, this BRAF-V600E mutation, although contributing to Erk1,2 phosphorylation, is not sufficient for sustained activation. Instead, full length CSPG4 and BRAF-V600E both appear to be required for sustained Erk 1,2 activation (193) and a CSPG4-specific mAb enhanced and increased the duration of the effects of a BRAF inhibitor in melanoma cells (194).

Transfection of CSPG4 stimulated melanoma cell motility in a scratch wound assay (193), an effect believed to be indicative of metastatic potential. Interestingly, CSPG4 stimulates α4β1-integrin-mediated adhesion and spreading, as well as FAK phosphorylation. Signaling through CSPG4 induces the recruitment and phosphorylation of p130cas indicating that CSPG4 signaling may intersect integrin-mediated signaling pathways even though it can signal independently of integrins (195). Interestingly, β1-integrin activation occurs as a result of CSPG4/NG2 phosphorylation and phosphorylation of different threonines trigger different β1-integrin-mediated events; either proliferation (Thr2314 phosphorylation) or motility (Thr2256 phosphorylation) (196).

Other evidence implicates CSPG4 in integrin-controlled cell activities. Chondroitin sulfate binds to the SG-1 site on α4 integrin subunits, and activation of this site is important for α4β1 binding to its ligand, the CS1 site on fibronectin (197). On melanoma, it is predominately chondroitin sulfate carried by CSPG4 that binds and activates the SG-1 site.

The chondroitin sulfate chain addition to CSPG4 also allows CSPG4 to interact directly with fibronectin through its heparin-binding domain. Ligand induced clustering of α4β1 causes the co-localization of CSPG4 and α4β1 (197). NG2/CSPG4 also associates with α3β1 via an interaction with galectin-3. Galectin-3 binds to N-linked oligosaccharides within the D3 domain of the CSPG4 core protein (198) and to oligosaccharides on β1 to form a complex that can be immunoprecipitated from human melanoma cell surfaces (167). It has been suggested that galectin-3 mediated clustering of NG2/CSPG4 and α3β1 leads to enhanced α3β1 signaling (167) and the promotion of melanoma invasion and migration through laminin containing extracellular matrices, because α3β1 selectively binds laminin and galectin-3 binds oligosaccharides on laminin.

Another mechanism by which CSPG4 facilitates melanoma metastasis is by its interaction with MMP-2. This complex comprises the inactive zymogen of the matrix metalloproteinase MMP-2, pro-MMP-2, which binds to the chondroitin sulfate chains of CSPG4. This interaction facilitates the generation of active MMP-2 (discussed later in this review) (199).

Collectively, the data suggest that CSPG4 acts as a scaffold at the cell membrane to facilitate the formation of molecular complexes that stabilize integrins and receptor tyrosine kinases, and localize active MMP-2 to the melanoma cell surface. The result of this is enhanced integrin signaling and ECM degradation, plus more effective growth factor activation of the RAS-RAF-MEK-Erk 1,2 pathway to increase cell proliferation and motility.

## Matrix Metalloproteinase-2

Matrix metalloproteinases are a family of zinc-dependent enzymes that degrade different ECM proteins (200). There are at least 26 different MMPs, which are classified into five groups according to their structure and substrate specificity – collagenases, gelatinases, stromelysins, membrane type MMPs (MT-MMPs), and others (200, 201). The constitutive gene expression of MMPs is low, but when the ECM is remodeled, whether for normal physiological or pathological processes, expression of these enzymes increases. The MMPs play a crucial role in physiological and pathological remodeling of the ECM during angiogenesis, wound healing, embryogenesis, and tumor metastasis (202). Degradation and remodeling of the ECM during melanoma metastasis allows tumor cells to invade surrounding ECM, spread via the lymphatic or vascular circulation, and extravasate into distant organs (200). The role of MMPs in tumor cell invasion is not limited to degradation of matrix components – additional substrates for MMPs include proteinases, proteinase inhibitors, other MMPs, growth factors, chemokines, cytokines, and cell surface proteins (203, 204). Thus, MMPs contribute to cell migration, proliferation, and apoptosis; and regulate tumor growth, vascularization, and spread (205).

The gelatinases, MMP-2, and MMP-9, are often over-expressed in malignant cancer. These enzymes degrade basement membrane proteins, such as collagen types IV, V, VII, X, and fibronectin. In melanoma, MMP-2 has frequently been associated with malignant progression and poor prognosis (200, 201, 206). A recent study using tissue microarray and immunohistochemistry of melanoma biopsies of primary and metastatic lesions as well as nevi concluded that MMP-2 expression is a prognostic indicator in primary but not metastatic lesions (201). This suggests that strong MMP-2 expression in the primary lesion contributes to the invasiveness of primary tumor cells, leading to metastases and poor survival outcomes. These findings are in accord with an earlier immunohistochemistry study of primary melanoma biopsy tissue. This study revealed that patients with a low number of MMP-2 positive cells (5–20%) in the tumor sample survived as well as those with an MMP-2 negative melanoma (10 year disease-specific survival rate of 79%), whereas patients with a primary tumor with high MMP-2 expression (>20% of tumor cells) had a 10-year disease-specific survival rate of 51% (207). The survival rate of this patient cohort declined further when proliferative activity of the tumor cells (indicated by Ki67 protein expression levels) and activation of apoptosis (revealed by p53 immunogenicity) were considered. Patients with primary melanoma having all three of these adverse factors had a 10-year survival rate of 28% (207). Interestingly, although MMP-2 and MMP-9 act on similar substrates, and are both expressed in melanoma, MMP-2 appears to be the better prognostic indicator (16, 207, 208).

Matrix Metalloproteinase-2 is synthesized and secreted as a 72 kDa pro-enzyme. It is activated primarily at the cell surface by proteolytic cleavage by membrane type 1 MMP (MT1-MMP/MMP-14); a process that is regulated by the concentration of tissue inhibitor of metalloproteinases-2 (TIMP-2). Activation of MMP-2 requires the formation of a ternary complex consisting of MT1-MMP, TIMP-2, and MMP-2. To form this complex, TIMP-2 first binds to MT1-MMP, and pro-MMP-2 then binds to TIMP-2. This facilitates cleavage of pro-MMP-2 by a neighboring active (TIMP-2 free) MT1-MMP, generating an intermediate 64 kDa MMP-2 fragment (205). This fragment then undergoes autocatalysis (209) or is further cleaved via the plasmin-plasminogen system to produce a fully active molecule (208). At high concentrations of TIMP-2, pro-MMP-2 activation is inhibited because TIMP-2 binds to both the pro-MMP-2 already complexed with MT1-MMP and to neighboring MT1-MMP molecules, so that pro-MMP-2 is unable to undergo cleavage and activation (205). However, the balance between free MT1-MMP and the MT1-MMP-TIMP-2 complex only partially determines the degree of MMP-2 activation (210).The relative amount of active and inactive MMP-2 also depends on the ratio of MT1-MMP and TIMP-2 expression and the quantity of TIMP-2 retained by low-affinity interactions with other plasma membrane molecules (211). Other members of the MT-MMP family (MT2-MMP and MT3-MMP) can also activate pro-MMP-2, but this does not involve TIMP-2. In addition, TIMP-1, -3, and -4 can regulate MT1-MMP activation of MMP-2 (212, 213).

Membrane proteins such as the claudins, αvβ3 integrin, and CSPG4 (discussed earlier) also participate in the activation of MMP-2. The association of these membrane glycoproteins with MMP-2 activation is of particular interest because αvβ3 integrin is often highly expressed on melanoma, claudin-1 expression levels increase with increasing thickness of the primary lesion (16) and CSPG4 is potentially a useful biomarker for melanoma. The chondroitin sulfate chains of CSPG4 have been shown to bind both pro-MMP-2 and MT3-MMP, an MT-MMP that is expressed on vertical growth phase melanoma and is important for melanoma invasion into collagen gels (199). CSPG4 appears to localize pro-MMP-2 in the vicinity of MT3-MMP, thereby assisting the generation of active MMP-2 (199), and this is likely to be important on melanoma cells where the surface density of MT3-MMP is relatively low. The tri-molecular complex comprising MT3-MMP, CSPG4, and pro-MMP-2 leads to activation of MMP-2 in the absence of TIMP-2 because structural features of MT3-MMP allow direct binding to the C-terminal domain of MMP-2 (199). Interestingly, claudin-1 binds to both MT1-MMP and pro-MMP-2 in regions that involve the catalytic domain of both enzymes, and this allows MT1-MMP to activate pro-MMP-2 in the absence of TIMP-2. In a similar mechanism to that described for CSPG4, it appears that claudin-1 localizes MT1-MMPs and pro-MMP-2 on the cell surface to produce local elevated concentrations of these enzymes, which enhances the activation of pro-MMP-2 (214). In melanoma cells, overexpression of claudin-1 is associated with increased activation of MMP-2; there is more MMP-2 associated with the cell surface than in non-transfected cells, and knockdown of claudin-1 in melanoma cells using siRNA decreases both the amount of active MMP-2 secreted and cell motility (215).

The role of αvβ3 in MMP-2 activation seems to be most important in the invasive growth phase of melanoma as expression of this integrin begins when melanoma cells switch from a horizontal to a vertical growth phase (216). A number of authors have reported data supporting the conclusion that αvβ3 binds active MMP-2 on the surface of melanoma cells (217, 218), others have found MMP-2 to be localized at the leading edge of migrating melanoma cells before αvβ3 (219), or that pro-MMP-2 did not bind αvβ3 (199). In the latter study the melanoma cells expressed MT3-MMP, not MT1-MMP. It is known that αvβ3 physically associates with MT1-MMP and the enzyme processes the integrin αv subunit into heavy and light chains connected by a disulfide bridge, which is the mature form. In cells lacking MT1-MMP, processing of αvβ3 occurs via another integrin convertase, like furin, but cleavage occurs at different sites and this mature αv chain is less able to promote adhesion and migration than the MT1-MMP processed αv chain (220).This and other data suggest the contribution of αvβ3 to MMP-2 activation depends on the co-expression of MT1-MMP. It has been reported that the MT1-MPP cleaved αvβ3 integrin can bind to the intermediate 64 kDa form of MMP-2 and enhance the autocatalytic step of the activation process to produce more of the mature MMP-2, as conversion of the intermediate to the mature form was low in the absence of αvβ3 (221).

Invadopodia, plasma membrane extensions enriched in cell-matrix adhesion molecules, actin-assembly regulators and proteases, form in the adhesive region of invasive tumor cells grown on an ECM. MT1-MMP traffics to these structures in cancer cells (222), suggesting that co-localization of αvβ3 with MT1-MMP and active MMP-2 concentrates adhesion molecules that bind matrix proteins with enzymes that degrade the matrix, thereby facilitating melanoma cell invasion. Moreover, αvβ3 dependent melanoma cell adhesion preferentially occurs on fibronectin fragments cleaved by MMP-2 rather than on intact fibronectin, and fibronectin fragments appear to promote αvβ3 recruitment into the invasive front of melanoma cells (219).

The conclusions from the *in vitro* studies are supported by *in vivo* data. In melanoma tissue sections, *in situ* zymography revealed MT1-MMP and secreted MMP-2 accumulate at the invasive front of melanoma cells, and the presence of functionally active MMP-2 is restricted to this region (223, 224). In another study of biopsies from patients with primary melanoma and patients with cutaneous or nodal metastases, MMP-2 expression was primarily in thick primary melanoma and in melanomas from patients who developed metastasis in the 3-year follow-up period (225). Thus, MMP-2 is very strongly associated with invading vertical growth melanomas. MMP-2 expression is not confined to tumor tissue as the surrounding stroma also synthesizes MMP-2, and in an experimental murine system MMP-2 expression was primarily attributed to the stroma (226), However, these data do not fit with the wealth of patient studies that suggest MMP-2 is a useful biomarker for melanoma.

## Conclusion

Most patients diagnosed with melanoma now present with thin lesions less than 1 mm thick and 90% of these patients will be cured by surgical excision. However, approximately 5% of these patients will develop metastatic melanoma and die within 10 years, despite no evidence of metastasis at the time of diagnosis. Using diagnostic criteria, there is no way to triage these patients into high and low risk groups, which limits our ability to direct screening and early treatment to those patients at higher risk of metastasis. Moreover, the treatment of metastatic melanoma has advanced little in the last three decades, with ipilimumab (a monoclonal antibody targeting CTLA-4 on T cells) and the BRAF inhibitor, vemurafenib, the only treatments to show an increase in overall survival and an extension of survival time, respectively. Unfortunately, ipilimumab often has significant side effects and is suitable for only a small proportion of patients. In addition, virtually all patients prescribed the BRAF inhibitor will develop clinical resistance and progressive disease. The reader is referred to a recent review on immunotherapy in advanced melanoma (227). Thus, there is an urgent need for additional prognostic markers and therapeutic targets. It is clear that multiple markers will be required to provide accurate prognostic information at diagnosis, and multiple parts of the metastatic pathway will need to be targeted to improve survival in patients with metastatic melanoma.

This review has focused on five molecules involved in melanoma metastasis – MCAM, Gal-3, CSPG4, MMP-2, and PAX-3. All of these molecules are expressed by a high proportion of primary and metastatic melanoma and have been described by others as biomarkers for melanoma. The word “biomarker” can be defined as: “A characteristic that is objectively measured and evaluated as an indicator of normal biological processes, pathogenic processes, or pharmacologic responses to a therapeutic intervention” (228). Our goal in this review has been to examine the expression patterns and functions of each of these molecules, with a focus on whether these “biomarkers” reveal the pathogenic processes of melanoma metastases. We believe that a good biomarker could also be a therapeutic target, and that examining the expression of a combination of molecules involved in different aspects of the metastatic process will provide better prognostic information compared to that obtained from a single biomarker.

**Figure 1:**
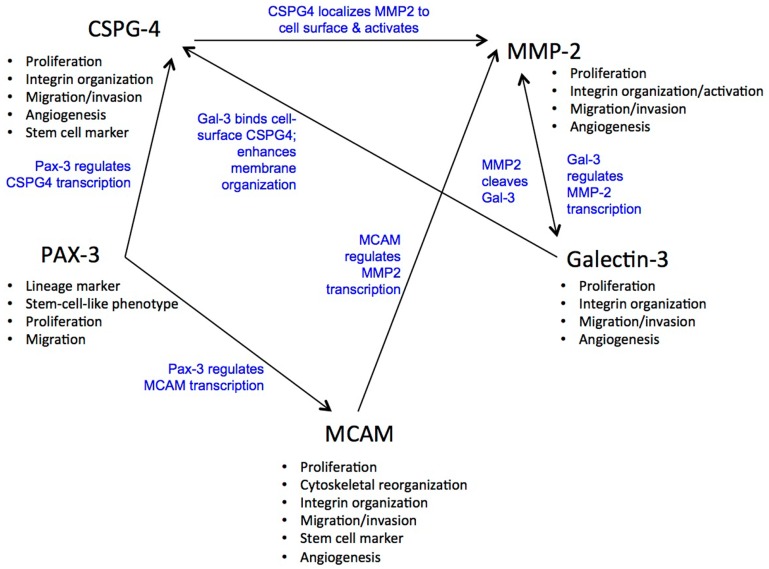
**Functional associations between MCAM, Gal-3, CSPG4, MMP-2, and PAX-3**.

In this review we have shown that these five molecules, although they have unique roles, both interact with each other and show similarities in their function. For example, both Gal-3 and PAX3 are anti-apoptotic, Gal-3 binds CSPG4 and Gal-1 binds MCAM. MCAM downstream signaling regulates the expression of MMP-2, nuclear Gal-3 up-regulates MMP-2 expression and MMP-2 cleaves Gal-3. MCAM, CSPG4, and Gal-3 are associated with angiogenesis and CSPG4 is involved with the activation of pro-MMP-2 on melanoma (relevant references are in the review). It will be interesting to see if Gal-3 can similarly bind MCAM as although both Gal-1 and Gal-3 bind glycosylation structures presented by core proteins the binding specificities of these two galectins differ. Gal-1 can recognize a range of different complex *N*-glycans, whereas Gal-3 recognizes poly-*N*-acetyllactosamine containing glycans that may be N- or O-linked (229). Figure 1 displays more of the cross connections that were revealed by the detailed examination of these five molecules.

It is particularly interesting that the combination of PAX3, MCAM, and CSPG4 is associated with less differentiated, motile cells of the melanocytic lineage and MCAM and CSPG4 are recognized stem cell markers. Indeed, the genes encoding these two stem cell markers are targets of PAX3 (68). The fact that the majority of metastatic melanoma express these stem cell markers, and when present, neither MCAM or CSPG4 is expressed by a minor population of cells within the melanoma leads one to think about rare cancer stem cells in melanoma. Interestingly, it has been demonstrated that approximately one in four cells from stage II, III, and IV melanomas obtained directly from patients are capable of developing tumors and moreover many markers are reversibly turned on and off *in vivo* (230). These findings directly question whether melanoma follows a cancer stem cell model and they also indicate that multiple biomarkers should be examined at each stage of melanoma progression for a reliable indication of prognosis.

**Figure 2:**
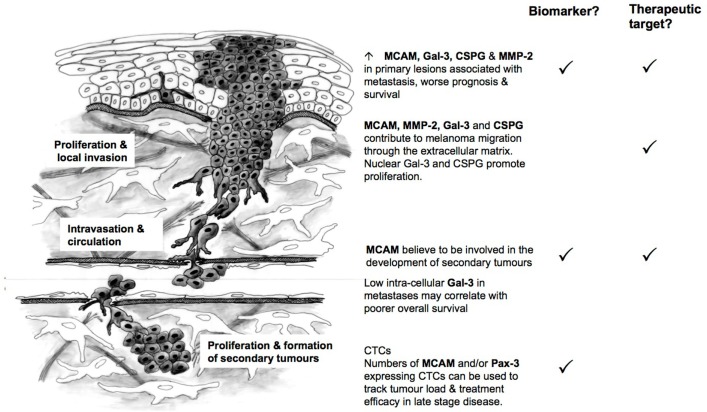
**MCAM, Gal-3, CSPG4, MMP-2, and PAX-3 as biomarkers and targets in melanoma metastasis**.

MCAM, MMP-2, and Gal-3 expression in primary melanoma have been linked to poorer overall survival (89, 90, 144, 206, 207) and could be used in combination with current prognostic indicators to identify patients at high-risk of recurrence (Figure 2). MCAM is believed to contribute to the later stages of metastatic spread (e.g., the formation of secondary tumors) (107), while MMP-2, and CSPG4 are likely to play a role earlier in the course of the disease. Gal-3 shows a bi-modal distribution – with increased intracellular expression early in disease progression and decreased expression in later metastatic lesions (144). This is due to Gal-3’s ability to act both as a transcriptional activator within the nucleus (147–150) and as a mediator between cell surface proteins (e.g., CSPG4, MCAM, integrins) and the ECM in the extracellular environment (161, 163, 167, 231). PAX-3 is expressed by all cells of the melanocytic lineage and is a key player in melanocyte development (36). However, it has recently been suggested that melanoma may be driven by cells with a less differentiated, highly motile phenotype and that PAX-3 may actively drive melanoma progression (57, 58). Currently, PAX-3 along with MCAM appears to be a useful biomarker for assessing tumor load and the effectiveness of treatment in later stage disease (55).

Although molecular biomarkers for cutaneous melanoma have received a lot of attention in recent years the introduction of one or more molecular biomarkers into clinical melanoma staging has lagged behind other cancers. This is partly due to the nature of the disease, and is compounded by the increasing diagnosis of melanoma from thin primary lesions, which leave no tissue for study outside of the standard clinical pathology procedures. In addition, some melanoma may recur many years after the original diagnosis, whereas others may recur within 5 years (17). We have highlighted throughout our review that currently there is no way of predicting which patients with thin melanomas are likely to relapse and when. The fact that cutaneous melanoma originates in melanocytes that have arisen from the neural crest and migrated to the skin is an additional difficulty, as this suggests normal melanocytes may have a molecular signature characteristic of an invasive phenotype. Therefore, the use of multiple markers will provide the best indicator of prognosis. Specifically, we believe that further study of a panel of markers, like those examined here, which have overlapping functions and are implicated at multiple stages of the disease process, may lead to the identification of a set of genes that can reliably assist in diagnosis and prognosis. Whether or not a combination of MCAM, MMP-2, CCPG4, PAX-3, and Gal-3 can identify those thin melanomas that comprise the 5% that will develop metastases at a later stage will require further studies of clinical material.

## Conflict of Interest Statement

The authors declare that the research was conducted in the absence of any commercial or financial relationships that could be construed as a potential conflict of interest.
